# Staging laparotomy for endometrial cancer in a patient with situs inversus totalis: A case report

**DOI:** 10.3892/ol.2014.2355

**Published:** 2014-07-16

**Authors:** EMIN USTUNYURT, TAYFUR CIFT

**Affiliations:** Department of Obstetrics and Gynecology, Bursa Şevket Yilmaz Research and Education Hospital, Bursa, Nilüfer 16120, Turkey

**Keywords:** situs inversus totalis, endometrial cancer

## Abstract

Situs inversus is a rare congenital anomaly in which the organs are transposed from their normal site to the opposite side of the body. To the best of our knowledge, this is the first study of staging laparotomy performed in a patient with endometrial carcinoma and situs inversus totalis (SIT). This study presents a patient with early endometrial carcinoma with SIT who underwent staging laparotomy. Total abdominal hysterectomy with bilateral salphingoophorectomy, omentectomy and pelvic para-aortic lymph node dissection was successfully performed without additional blood loss and time. The number of retrieved lymph nodes was 47. No abnormal course of blood vessels except for the right/left inversion was found. The postoperative course was favorable and the patient was discharged eight days after surgery.

## Introduction

Situs inversus totalis (SIT) is a rare congenital condition characterized by a mirror-image transposition of the abdominal and thoracic viscera, its incidence accounts for 1/8,000 to 1/25,000 of the worldwide population (1). Occasionally, patients with a combination of this condition and malignant tumors have been encountered.

Previous studies on the surgical procedures for patients with SIT have documented technical difficulties, as the anatomy is abnormal (2–4). Surgery for patients with SIT and cervical cancer has been previously reported; however, to the best of our knowledge, there has been no report of surgery for endometrial cancer in patients with SIT in the English literature to date (5). This study presents a patient with endometrial cancer with SIT who underwent staging laparotomy.

Written informed consent was obtained from the patient for publication of this case report and accompanying images.

## Case report

A 44-year-old woman visited a local hospital reporting abnormal vaginal bleeding for six months. Endometrial biopsy revealed an endometrioid adenocarcinoma and the patient was referred to Bursa Şevket Yılmaz Research and Education Hospital (Bursa, Turkey) in March, 2013 for further evaluation and surgical treatment.

**Figure 1:**
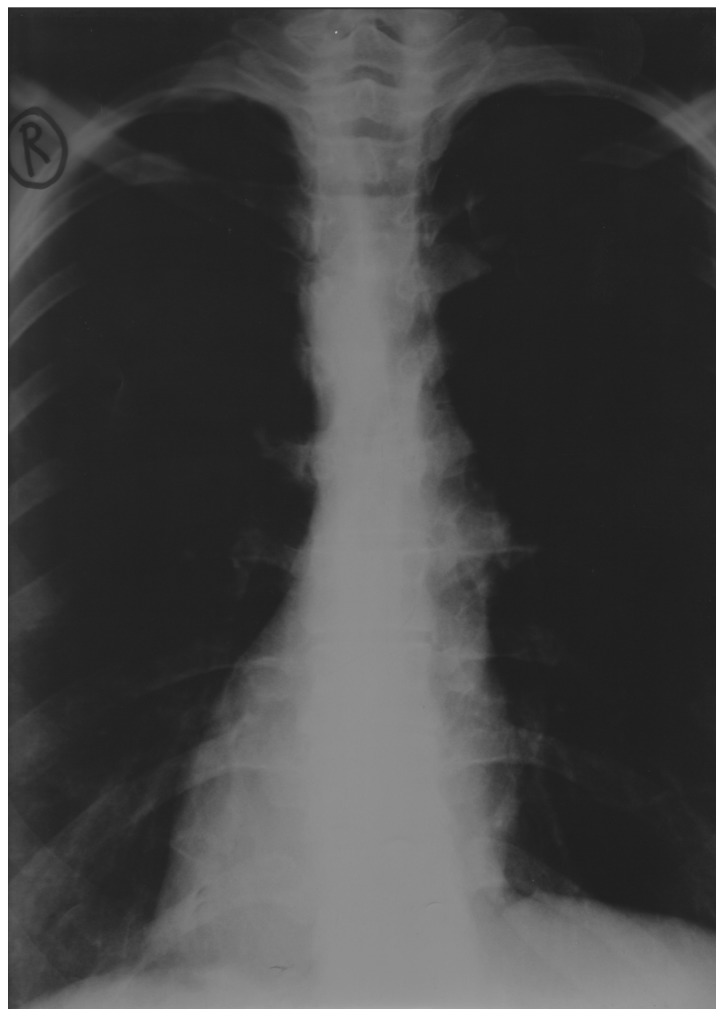
Chest radiography of the patient. Dextrocardia and a right subphrenic gastric bubble are evident.

A standard preoperative chest radiograph revealed dextrocardia with the stomach bubble situated on the right (Fig. 1). Ultrasound and computed tomography (CT) abdominal scanning revealed situs inversus. Abdominal CT showed that all organs inside the abdomen were inversely positioned without enlarged lymph node, distant metastasis and abnormal course of vascularity. No other physical abnormalities were noted. Routine laboratory tests yielded normal results and the serum levels of carbohydrate antigen (CA) 19-9 and CA 125 were normal.

**Figure 2:**
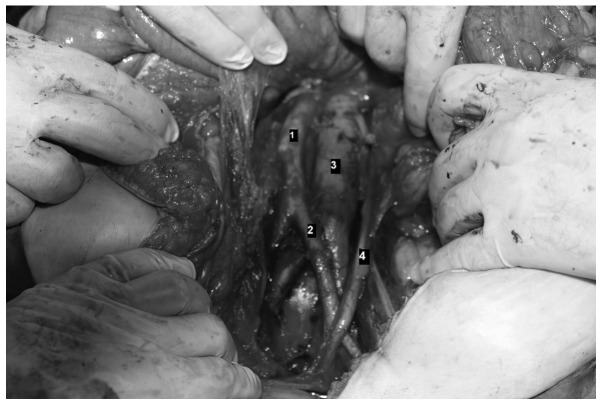
Intraoperative image of major vessels. 1) Abdominal aorta, 2) left common iliac artery, 3) inferior vena cava and 4) left ureter.

In March, 2013, a staging laparotomy was performed. Following administration of general anesthetic, the patient was placed in a lithotomy position. The operator was situated on the left and the first assistant was on the right (usual side of surgery at the Bursa Şevket Yılmaz Research and Education Hospital). The abdomen was explored through a midline incision. As expected, complete transposition of the viscera was observed, the stomach and spleen were lying on the right side, and the gall-bladder, the large lobe of the liver and the caecum and appendix were situated on the left. There was no macroscopic serosal invasion and no evidence of ovarian, hepatic or peritoneal metastases. Following explorative total abdominal hysterectomy, bilateral salphingoophorectomy was performed. Pelvic lymphadenectomy was performed in the usual manner. An incision in the right lateral paracolic gutters with subsequent medial reflection of the right colon was made to remove the right para-aortic lymph nodes. An incision was then made in the peritoneum overlying the left common iliac artery (the right in an orthotopic patient). The incision was performed over the aorta to the level of the duodenum. Using blunt dissection, the left ureter and left ovarian vessels were identified and mobilized laterally. The lymphatic tissue lateral to the left common iliac artery was removed. The lymphatic packages were retrieved along the left side of the aorta and the anterior surface of the inferior vena cava up to the level of the renal veins (Fig. 2).

The position of the operator and assistant did not differ from those in orthotopic patients. Total abdominal hysterectomy, bilateral salphingoophorectomy and lymphadenectomy in our patient were successfully performed by careful consideration of the mirror-image anatomy. The total operation time was 3 h and 15 min, and the total blood loss was 1,280 ml.

Histological analysis of the resected specimen confirmed the diagnosis of well-differentiated adenocarcinoma of the endometrium infiltrating more than half of the myometrium. Surgical staging showed no distant metastasis or metastatic lymph nodes and the tumor was classified as stage 1B. The postoperative course was uneventful and the patient was discharged from hospital eight days after surgery. Following discharge, the patient received adjuvant radiotherapy at the referred clinic (Department of Medical Oncology, Ali Osman Sönmez Oncology Hospital, Bursa, Turkey). Regarding the myometrial invasion, complementary radiation was administered. Follow-up examinations revealed that the patient was fit without any evidence of recurrence six months after surgical treatment.

## Discussion

SIT is inherited in an autosomal recessive manner and requires great care due to abnormal anatomy. If the surgical resection of any organ is required, it is important to understand the association between the organ and its vascular structure (6).

An increased risk of cardiac, splenic and hepatobiliary malformations are found in patients with SIT (7,8). Although rare malignant neoplasms have been reported; this abnormality is not considered to be a malignant entity (7).

In the present case, surgery itself presented marginal difficulties. With SIT, abnormal vascularization of the arteries and veins is common (9); therefore, the preoperative confirmation of any abnormal vascularization is important. The vascularity of our patient was examined through abdominal CT scanning, which confirmed no evidence of abnormal vascular malformation. In one study, CT alone was considered to be insufficient for determining the vessel anatomy, however, 3D reconstruction of an abdominal CT angiography image was reported to obtain an improved result (10). During surgery the surgeon and assistant may reverse positions, but is not likely to gain any advantage. As a result, it is more important to understand the anatomy and abnormal vascularization prior to surgery (11). To prevent surgery-related complications, such as intraoperative bleeding or organ injury, confidence in vascular anatomy and procedural carefulness are mandatory. Therefore, surgery may be performed with a sufficient number of retrieved lymph nodes and without additional blood loss and time.

In conclusion, a review of the English literature did not reveal any cases of endometrial carcinoma complicating complete situs inversus. To the best of our knowledge, this is the first study on staging laparotomy for an endometrial cancer patient with SIT. Due to the frequency of associated malformations of transposed organs and vascular anatomical variations that make surgical management difficult, special attention should be paid to diagnosis and preoperative staging.
